# Late eating, blood pressure control, and cardiometabolic risk factors among adults with hypertension: results from the Korea National Health and Nutrition Examination Survey 2010–2018

**DOI:** 10.4178/epih.e2021101

**Published:** 2021-11-24

**Authors:** Jee-Seon Shim, Hyeon Chang Kim

**Affiliations:** 1 Department of Preventive Medicine, Yonsei University College of Medicine, Seoul, Korea; 2 Department of Internal Medicine, Yonsei University College of Medicine, Seoul, Korea

**Keywords:** Meal time, Circadian rhythm, Hypertension, Blood pressure, Cardiometabolic risk factors

## Abstract

**OBJECTIVES:**

Despite growing concerns regarding the timing of eating, little is known about the association between late eating and health. This study aimed to investigate whether late eating is associated with blood pressure (BP) control and cardiometabolic risk factors among Korean adults with hypertension.

**METHODS:**

Data from the Korea National Health and Nutrition Examination Survey 2010–2018 were used. Adults with hypertension aged 30–79 years (n=13,361) were included in this study. Dietary intake and information on meal timing were assessed using 1-day 24-hour recall. Late eating was defined as after the median midpoint between the times of the first and the last eating episode during the recall day. Logistic and linear regression models were used to estimate the associations of late eating with BP control and cardiometabolic risk factors.

**RESULTS:**

Among late eaters, there were more men than women. Compared to early eaters, late eaters were younger, had a higher body mass index (BMI) and unhealthier habits, and their overall dietary quality score was lower. A negative association between late eating and BP control was found in a univariate model (odds ratio [OR], 0.82; 95% confidence interval [CI], 0.94 to 1.12), but this association disappeared after adjustment for confounders (OR, 1.03; 95% CI, 0.94 to 1.12). Late eating was independently associated with higher BMI (p=0.03) and blood triglyceride concentration (p<0.01).

**CONCLUSIONS:**

Our results do not support a link between late eating and BP control among adults with hypertension, but suggest that late eating is associated with cardiometabolic risk factors.

## INTRODUCTION

Hypertension is a major public health burden worldwide [1]. Raised blood pressure (BP) is a leading cause of cardiovascular disease and mortality, but is also the strongest modifiable risk factor [2–4]. On the basis of scientific evidence of the beneficial effects of healthy lifestyle on BP control as well as overall health, lifestyle modifications such as a healthy diet and increased physical activity have been recommended as a first-line strategy to reduce BP [5,6]. Even for patients on antihypertensive medication, non-pharmacological treatment is still strongly recommended as a combination treatment [3,5,6].

Thus far, data on the effects of diet on BP have been acquired for some aspects of diet, such as nutrient intake (e.g., dietary fiber, polyunsaturated fats, potassium, and vitamins), food group consumption (e.g., fruits and vegetables, fish, and nuts), and dietary patterns (e.g., the Dietary Approaches to Stop Hypertension diet and the Mediterranean diet) [7–9]. Based on this evidence, health professionals have made recommendations about food consumption—both in terms of what and how much to eat—in order to prevent hypertension and to reduce BP [5,6]. However, both the prevalence of self-reported diet management and compliance with dietary recommendations are relatively low compared to compliance with antihypertensive medication or other lifestyle modifications, such as smoking cessation [10–15]. Although the BP-lowering effects of reduced sodium intake and healthy diet are well established in both normotensive and hypertensive people [9,16,17], people have still considerable difficulties preparing meals according to the recommendations, managing their diet in special situations (e.g., eating out), and modifying old habits, among other factors [16,18–20]. For successful dietary modification, there is a need for new strategies to lower potential barriers to diet management, coupled with efforts to better understand the factors influencing dietary compliance.

In recent years, interest has grown in the timing of eating beyond traditional dietary approaches focusing on foods and nutrients. A longer period of food intake each day, irregular eating, and eating at the wrong time are associated with more energy intake and poor diet quality [21–24]. Previous studies have shown that late eating was associated with lower efficacy of weight loss interventions than early eating [25], and that early time-restricted eating reduced appetite [26] and improved insulin sensitivity, BP, and oxidative stress [27]. Erratic eating patterns have been found to adversely influence obesity, diabetes, metabolic syndrome, and cardiovascular diseases [21,25,28,29]. However, that evidence was obtained from carefully controlled feeding studies with specific populations such as pregnant women or obese adults [21,22,25,27]. There is little evidence available on characteristics related to eating time, BP control, and cardiometabolic health among adults with hypertension in a real-world setting. Thus, this study aimed to investigate whether late eating is associated with BP control and cardiometabolic risk factors among Korean adults with hypertension.

## MATERIALS AND METHODS

### Data source and study population

This study used data from the Korea National Health and Nutrition Examination Survey (KNHANES) 2010–2018, which is an ongoing surveillance system conducted by Korea Disease Control and Prevention Agency (KCDA). This nationwide cross-sectional survey provides information on health and nutritional status.

In the KNHANES 2010–2018, there were 38,794 adults aged 30–79 years who had information on both BP and dietary intake. Among them, we excluded pregnant and lactating women (n=539) or individuals for whom inaccurate information was obtained regarding the time of each eating episode, e.g., “do not remember” or “all morning” (n=270). Among the remaining adults (n=37,987), we included adults with hypertension who took antihypertensive medicine or had systolic BP of ≥140 mmHg or diastolic BP of ≥90 mmHg. Thus, a total of 13,361 adults with hypertension (6,244 men and 7,117 women) were retained in the present study.

### Dietary intake and definition of late eating

Dietary intake was assessed using 1-day 24-hour recall. Trained dietitians collected information on dietary intake and habits. Participants were requested to report when, where, what, and how much they ate and drank on the recall day. Both the time of the day when they consumed foods and beverages (e.g., 07:40) and each eating episode (e.g., breakfast, lunch, dinner, and snack) were collected. If homemade dishes were consumed, the recipe was collected from the person who prepared the dishes. The amount of the foods and beverages participants consumed was reported using measuring aids (e.g., 2-dimensional drawings, rulers, measuring cups and spoons), and then converted into weight (g) by using the database for volume and weight of foods. All dietary recall procedures were performed using a standardized protocol involving the multiple-pass method. Dietary intake of food and nutrients was calculated using Korean food composition data developed for the KNHANES [30–32]. In addition to dietary intake, the raw data of the KNHANES included the overall diet quality score calculated using the Korean Healthy Eating Index (KHEI). The KHEI evaluates how well an individuals’ diet complies with the dietary guidelines for Koreans. The KHEI consists of 14 components (8 for adequate intake, 3 for moderate intake, and 3 for balanced diet) and the score ranges from 0 to 100, with a higher score reflecting better diet quality [14,33]. More details are available elsewhere [14,32–35].

Late eating was defined by the midpoint between the times of the first and the last eating episode during the recall day, based on the operational definition applied in previous studies [25]. The median midpoint of eating episodes in our study population was 13:45; therefore, participants were divided into 2 groups according to the midpoint of their eating episodes as early eaters (before 13:45) and late eaters (after 13:45), respectively. The frequency of meals and snacks was defined by the number of eating episodes. The proportion of dietary energy intake from meals and snacks for each participant was calculated.

### Blood pressure control and cardiometabolic risk factors

BP was measured 3 times at 5-minute intervals after resting for more than 5 minutes in a sitting position. The mean of the last 2 measurements was used in the present study. Controlled BP was defined as systolic BP <140 mmHg and diastolic BP <90 mmHg. Body weight and height were measured to the nearest 0.1 kg and 0.1 cm, respectively. Body mass index (BMI) was calculated as body weight divided by height squared (kg/m^2^). Waist circumference was measured to the nearest 0.1 cm at the midpoint between the lower rib cage and the iliac crest. Blood was collected after fasting for more than 8 hours. Fasting blood glucose, hemoglobin A1c, total cholesterol, triglycerides, and high-density lipoprotein-cholesterol were measured. All procedures of the health examination survey were performed by trained staff according to standardized protocols. Clinical laboratory tests were conducted in central certified laboratories. Further details on health examination are described elsewhere [30,34].

### Other variables

Information on household income, occupation and shift work, health-related behaviors, past history of diseases, and medication was collected via a health interview survey. Household income was defined as the family income adjusted by the number of family members. The quartiles of monthly household income defined by the KNHANES were used in this study. Information regarding work time was obtained through the responses “working during the day (6 a.m. to 6 p.m.)” or “at different times.” In our study, those who worked at different times (e.g., night workers or 24-hour shift workers) were defined as shift workers, and the rest—including day workers and the unemployed—as non-shift workers. Smoking, drinking, and physical activity were assessed based on self-reports. Current smoking was defined as ongoing smoking behavior at the time of the survey and having smoked more than 5 packs of cigarettes in one’s lifetime. Current drinking was defined as drinking at least once a month during the last year. Physical activity was surveyed in every survey year, but the same questionnaire was not applied every year. However, information on walking was collected using the same question and protocol. Thus, the number of days walked was used as information regarding physical activity. History of diseases and information regarding disease treatment were collected through interviews. Comorbid status was defined based on the self-reported presence of any diagnosis of diabetes, dyslipidemia, stroke, myocardial infarction, or angina pectoris. Taking BP-lowering drugs on more than 20 days a month was defined as high adherence to antihypertensive medication. Details on data collection and health interview survey are available elsewhere [30].

### Statistical analysis

Descriptive statistics on the time of eating episodes, demographic and health-related characteristics, and dietary profiles were presented as mean±standard deviation (SD) or proportion (%). Differences in variables between late eaters and early eaters were analyzed using the Student t-test for continuous variables and the chi-square test for categorical variables.

The associations of late eating with BP control in adults with hypertension were examined using 3 logistic regression models. Model 1 was used to estimate the crude odds ratio (ORs) and the corresponding 95% confidence intervals (CIs) of late eating for controlled BP compared with early eating. Model 2 was used to estimate the adjusted OR after adjustment for gender, age, shift work, smoking, drinking, walking, BMI, comorbid status, and antihypertensive medication. Model 3 further adjusted for total energy intake and overall diet quality (i.e., the KHEI score) plus the variables included in model 2. The KHEI has been included only in the raw data of the KNHANES since 2013, and thus model 3 was performed in a smaller population (n=8,493). We also performed multiple logistic regression analyses by gender (men or women), age (30–49, 50–64, or ≥65 years), shift work (yes or no), smoking (yes or no), drinking (yes or no), walking (<3.5 or ≥3.5 d/wk), obesity (BMI <23.0 or ≥23.0 kg/m^2^), comorbid status (yes or no), adherence to antihypertensive medication (high or low), overall diet quality (KHEI score <65.8 or ≥65.8, using the study population mean), and whether the diet on the recall day was the same as usual or not. In subgroup analyses, multiple logistic models were adjusted for other potential confounders.

To evaluate differences in cardiometabolic risk factors between late eaters and early eaters, we presented data as means±SDs by group and performed linear regression analyses. Crude models and adjusted models were applied, respectively. In multiple linear regression models, we adjusted variables included in model 2 of multiple logistic regression analyses as mentioned above. The results were presented as β coefficients and p-values. The data were analyzed using SAS version 9.4 (SAS Institute Inc., Cary, NC, USA). All statistical tests were 2-sided, and p-values <0.05 were considered to indicate statistical significance.

### Ethics statement

All survey protocols and procedures were approved by the Institutional Review Board of the KCDA (2018-01-03-P-A, 2018-01-03-C-A). Informed consent was obtained from each participant. Further details on the KNHANES are described elsewhere [30,34].

## RESULTS

### Time of eating episodes and demographic and health-related characteristics

Among 13,361 adults with hypertension, 53.3% were women and the mean age was 61.8 years (Table 1). On average, the first eating episode was at 07:47 (hh:mm) and the last was at 19:43, and the median midpoint of eating time in the day was 13:45. The mean times of late eaters for all eating episodes were delayed compared with early eaters. The mean midpoint of the eating period of later eaters was nearly 2 hours later than that of early eaters. Later eaters were more often men, were younger, had a higher income, had a higher BMI, were more often shift workers, smokers and drinkers, and were less physically active than early eaters. Among all adults with hypertension, 71.4% were already aware of their hypertension, two-thirds took antihypertensive medication over 20 days per month, and 43.3% were diagnosed with any cardiometabolic diseases such as diabetes, dyslipidemia, stroke, myocardial infarction, or angina pectoris. Later eaters had relatively lower awareness of hypertension and antihypertensive treatment than early eaters.

### Dietary intake, the number of eating episodes, and overall diet quality

Table 2 shows the differences in dietary profiles between late eaters and early eaters. Late eaters had more eating episodes and more energy intake than early eaters. Their meal frequency was slightly lower, but the snack frequency was much higher, and the proportion of snacking to total energy intake was higher than for early eaters. Late eaters also had lower KHEI scores than early eaters. Although some of the 14 KHEI components (i.e., meat/fish/eggs/legumes, milk/milk products, carbohydrate, and total fat) presented higher scores in late eaters and some (i.e., fruits, sweets and beverages, and balanced energy intake) showed no differences between late eaters and early eaters, all other component scores were higher in early eaters. Thus, late eaters had much poorer dietary profiles than early eaters.

### Association of late eating with blood pressure control

**Figure 1:**
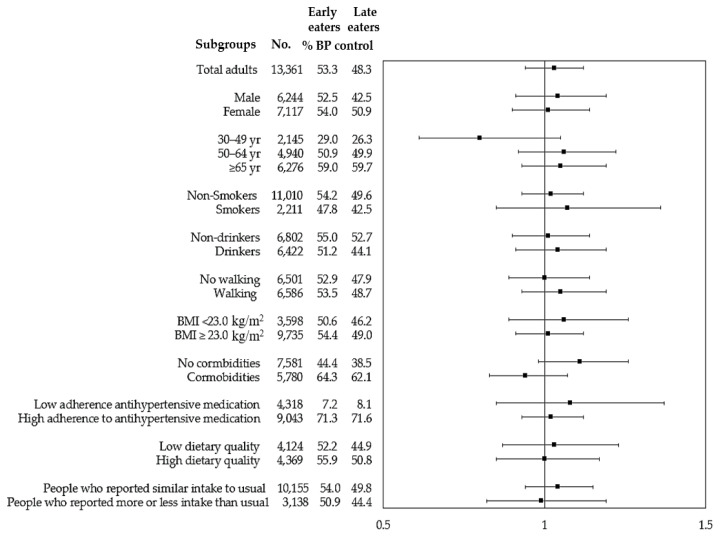
Subgroup analyses for associations of late eating with blood pressure control in hypertensive adults. ORs after adjustment for gender, age, shift work, smoking, drinking, walking, BMI, comorbid status, and adherence to antihypertensive medication. Data are presented as OR and 95% CI. The Korean Healthy Eating Index was available for the KNHANES 2013–2018 participants, and thus subgroup analyses for diet quality were performed using data of adults in 2013–2018 (n=8,493). OR, odds ratio; CI, confidence interval; KNHANES, Korea National Health and Nutrition Examination Survey.

Among our study participants, 50.8% had controlled BP (data not shown). Table 3 shows the ORs of late eating for BP control compared with early eating. Late eaters had a significantly lower OR for BP control in model 1 (0.82; 95% CI, 0.77 to 0.88), but this association disappeared after adjustment for other potential confounders in model 2 (OR, 1.03; 95% CI, 0.94 to 1.12) and model 3 (OR, 1.02; 95% CI, 0.90 to 1.15). Similar associations were found in subgroup analyses (Figure 1). Overall, an independent association between late eating and BP control was not found after adjusting for potential confounders.

### Associations of late eating with cardiometabolic risk factors

Table 4 shows the associations between late eating and cardiometabolic risks, including BP, identified through linear regression analyses. In simple regression models, compared with early eaters, late eaters had significantly higher diastolic BP (3.20 mmHg), BMI (0.43 kg/m^2^), waist circumference (0.43 cm), triglycerides (14.70 mg/dL), and total cholesterol (3.50 mg/dL). After adjustment for potential confounders, the associations of late eating with BP, waist circumference, and total cholesterol disappeared. However, late eating still had a significant association with higher BMI (0.13 kg/m^2^, p=0.03) and triglycerides (6.43 mg/dL, p<0.01). We also investigated associations between the midpoint time of the eating period during the day and cardiometabolic risk factors in the same multivariate models. The midpoint of the eating period had no significant association with systolic/diastolic BP or other cardiometabolic risk factors, but each 10-minute delay in the midpoint of the eating period was associated with a 1.05 mg/dL increase in the blood triglyceride concentration after adjustment for potential confounders (data not shown).

## DISCUSSION

Using data from the KNHANES 2010–2018, we examined the associations of late eating with BP control and cardiometabolic risk factors among Korean adults with hypertension. In our study, late eating showed a negative association with BP control in a univariate model, but this association disappeared after adjustment for potential confounders. Late eating was significantly associated with higher BMI and a higher blood concentration of triglycerides, independent of potential confounders.

There have been few studies on meal timing, and none have focused on Koreans. Our study found that the average time of the first and last food intake of Korean adults with hypertension was 07:47 and 19:43, respectively, and the midpoint of the eating period was 13:45. In a study of overweight and obese adults in Spain [25], information on eating time was collected using questionnaires. The mean breakfast and dinner times were 08:31 and 21:21, respectively, and the median midpoint between breakfast and dinner was 14:54. Another study of healthy Indian adults showed that overall the median breakfast and last intake times were 06:58 and 22:45, respectively [36]. Our study participants had a slightly shorter eating duration and earlier dietary intake than participants in other studies [25,36].

Although there was no independent association between late eating and BP control among our study population of adults with hypertension, our findings concerning late eating, higher BMI, and higher triglyceride concentrations are consistent with previous studies on the detrimental effects of late eating on cardiometabolic risks and cardiovascular diseases [21,25,29,37,38]. Later circadian timing of food intake is reportedly associated with increased BMI and percentage of body fat, regardless of more traditional risk factors (e.g., the amount or content of dietary intake and physical activity) [25,37]. Late eaters had a higher blood concentration of triglycerides, lower insulin sensitivity, a higher concentration of leptin in the morning, and more obesogenic behaviors (e.g., eating when stressed, eating while watching TV, etc.) than early eaters [25]. The timing and distribution of dietary intake during the day seem to be related to human health. A British birth cohort showed that high energy intake late in the evening was associated with higher hypertension incidence and greater increases in BP over 10 years [29]. A carefully controlled inpatient study reported that nighttime eaters gained more weight than non-nighttime eaters after adjustment for baseline weight and follow-up time [21]. In a weight loss intervention trial, later eaters had a lower success rate and a lower rate of weight loss than early eaters [25].

In addition to later circadian timing of eating, there is evidence supporting the beneficial influence of shortening eating duration and early eating on human health [26,27,39,40]. Controlled feeding trials found that early time-restricted feeding (eating from 08:00 to 14:00 [26] and 6-hour feeding with dinner before 15:00 [27]) increased fullness and decreased the desire to eat [26], increased fat oxidation [26], and improved BP [27], insulin sensitivity [27], oxidative stress levels, and metabolic flexibility [26,27]. In a trial that only shortened eating duration (10–11 hours) for overweight adults with prolonged eating periods (>14 hours), subjects experienced body weight reduction, expressed more subjective sleep satisfaction, and reported feeling more energetic. Eating time also seems to be related to the composition and function of the human gastrointestinal microbiota [40]. These time-based dietary interventions usually focus on limiting the eating hours in the day (e.g., shortening the eating period or shifting the eating window to early in the day) without intentionally altering dietary quality or quantity. Time-based dietary approaches appear to be relatively easy to follow and adapt. In clinical feeding trials, time-restricted interventions not only have a high level of compliance and a low dropout rate [27,41], but also have few or no adverse effects [26,27,41], and thus have been proposed as a sustainable and effective strategy for dietary modification.

Several mechanisms may explain how time-related dietary characteristics, including late-eating, affect overall health [27,37,39,42–44]. One possible explanation is that early time-restricted eating may decrease dietary energy intake, even if people do not pay attention to calorie intake [39,43]. A decrease in calorie intake induces a decrease in body weight, thereby improving health conditions [43]. However, later circadian time-of-eating appears to play a role in body composition even after controlling for total calorie intake and other possible confounders [37]. Sutton et al. [27] found that early time-restricted feeding improved insulin sensitivity, BP, oxidative stress, and appetite even without weight loss, in their randomized, crossover, isocaloric and eucaloric controlled feeding trial. This impact of eating time on human health has been explained by several other mechanisms [42,45,46]. Previous studies found that circadian rhythms may regulate human metabolism (e.g., thermic effect of food, gluconeogenesis, glycolysis, protein or lipid synthesis, lipid oxidation, and mitochondrial function), maintain homeostasis, and also coordinate the process of nutrition [42]. This suggests that humans are optimized for food intake in the morning rather than later in the day, and that eating at the wrong time (i.e., eating at times misaligned with the circadian rhythm) can lead to circadian disruption, contributing to metabolic dysfunction and adverse impacts on health in spite of no change in food intake or physical activity [42,44]. Disrupted circadian rhythms are found in later life and also in disease conditions [46]. Scheduled meals (i.e., right-time eating aligned with the circadian rhythm) can entrain the circadian system and restore circadian rhythms [46]. However, further studies are still required in order to better understand the link between eating time and human health, because there is not enough information regarding which eating time periods and lengths of eating times are more beneficial for human health.

In Korea, one-third of adults aged 30 years or older are estimated to have hypertension. The rates of treatment and control of hypertension rapidly improved relative to the past, but there has been no substantial change for the last decade [47]. The increased control rate of hypertension may be due to increased antihypertensive drug treatment [47]. The low proportion of dietary management [14] and the low level of dietary adherence to guidelines for hypertension prevention and treatment are still problematic [16]. Moreover, our previous study found a distinct gap between normotensive adults’ attitudes towards hypertension treatment and the current health-related behaviors of adults with hypertension [16]. Although pharmacological treatment is a major contributor to reducing BP, dietary modification can also improve cardiovascular health [43]. Thus, efforts are needed to seek an easy-to-adapt and promising strategy for diet modification.

This study is the first attempt to show the characteristics of the eating time of Korean adults with hypertension and to examine the associations of eating time with BP control and cardiometabolic risk factors. We used the data from KNHANES, a nationwide study, and thus were able to include diverse adults with hypertension residing across Korea. We believe our findings are meaningful because they show an unfavorable association between late eating and some cardiometabolic health factors, even after adjustment for confounders such as pharmacological treatment. Despite these strengths, this study has some limitations to be considered. First, this study analyzed cross-sectional data. Thus, our findings do not demonstrate a causal relationship between late eating and BP control and cardiometabolic risk factors among adults with hypertension. Second, we defined late eating by using 1-day 24-hour recall data, which may not reflect individuals’ usual eating patterns in terms of time. To partly overcome the limitation of single-day dietary assessments, we additionally performed the same analyses only for adults with hypertension who reported that the amount of food they ate on the recall day was similar to the amount of food they usually ate (Supplementary Material 1), and the associations between late eating and cardiometabolic risk factors remained unchanged. Nonetheless, caution is still needed when interpreting these results. Lastly, although 24-hour recall data of the KNHANES contain information on the time when each participant consumed food, the dietary assessment of the KNHANES was originally intended to measure the dietary intake of foods and nutrients, not to capture the time of the day in detail. Thus, our data lack details on eating time, such as time-stamped ingestion data, which recent studies have collected using innovative smartphone apps [36,39].

In conclusion, this study did not find an independent association between late eating and BP control among Korean adults with hypertension, but found an inverse association between late eating and cardiometabolic health, independent of confounders. Our findings support the suggestion that a time-based dietary approach can be used as a useful strategy to improve the prognosis of adults with hypertension. Further research is needed to clarify the causal relationship between late eating and health and to understand the impact of other attributes related to meal timing (e.g., eating period and the eating time window) on health.

## Figures and Tables

**Table 1:** Time of eating episodes and general characteristics according to late eating in adults with hypertension

Characteristics	Total (n=13,361)	Early eaters (n=6,680)	Late eaters (n=6,681)	p-value^1^
Timing of eating occasions, hh:mm				
First intake	07:47±01:49	06:52±01:32	08:41±01:37	<0.001
Breakfast (n=11,947)^2^	07:56±01:15	07:29±01:10	08:27±01:08	<0.001
Lunch (n=12,382)^3^	12:45±01:07	12:34±01:03	12:56±01:08	<0.001
Dinner (n=12,661)^4^	18:48±01:31	18:21±01:38	19:14±01:16	<0.001
Last intake	19:43±01:44	18:40±01:22	20:46±01:22	<0.001

Midpoint of eating duration, hh:mm	13:45±01:18	12:46±00:47	14:44±00:55	<0.001

Gender (women)	53.3	54.4	52.1	0.008

Age, yr	61.8±11.5	64.5±10.3	59.1±12.0	<0.001

Income (highest quartile)	20.5	16.8	24.3	<0.001

Shift work (yes)^5^	8.4	7.7	9.1	0.003

Body mass index, kg/m^2^	25.1±3.4	24.9±3.3	25.3±3.5	<0.001

Current smoking (yes)	16.7	14.7	18.7	<0.001

Current drinking (yes)	48.6	45.4	51.7	<0.001

Physical activity (no. of days of walking)	3.7±2.7	3.8±2.7	3.6±2.7	0.003

Awareness of hypertension (yes)^6^	71.4	75.1	67.7	<0.001

Adherence to antihypertensive medication (high)^7^	67.7	72	63.4	<0.001

Comorbid status (yes)^8^	43.3	44.9	41.6	<0.001

Values are presented as mean±standard deviation or %.

1 Student t-test and the chi-square test.

2 Only for those who had breakfast.

3 Only for those who had lunch.

4 Only for those who had dinner.

5 Shift work included all types of shift work except day workers and those who were unemployed.

6 Awareness of hypertension was defined as physician-diagnosed hypertension.

7 Adherence to antihypertensive medication was defined as high (taking BP-lowering drugs for 20 days per month) or low (less than 20 days per month).

8 Comorbid status was defined as any presence of diabetes, dyslipidemia, stroke, myocardial infarction, or angina pectoris.

**Table 2:** Dietary profiles according to late eating in adults with hypertension

Variables	Total (n=13,361)	Early eaters (n=6,680)	Late eaters (n=6,681)	p-value^1^
Dietary intake				
Energy intake, kcal/d	1,889±840	1,826±796	1,952±878	<0.001
Carbohydrates (% of TE)	70.3±11.2	71.8±10.8	68.9±11.3	<0.001
Protein (% of TE)	14.1±4.2	13.8±4.1	14.3±4.3	<0.001
Fat (% of TE)	15.6±8.7	14.4±8.3	16.8±8.9	<0.001
Sodium, mg/d	3,885±2,858	3,731±2,733	4,039±2,969	<0.001

Frequency of eating occasions (total)	5.3±1.8	5.1±1.7	5.4±1.8	<0.001
Meals	2.8±0.5	2.8±0.4	2.7±0.5	<0.001
Snacks	2.5±1.7	2.3±1.7	2.7±1.7	<0.001

Meal energy density (% of TE)				
Meals	82.9±15.1	84.5±14.8	81.2±15.3	<0.001
Snacks	17.1±15.1	15.5±14.8	18.8±15.3	<0.001

Healthy eating index (score)^2^	65.8±12.4	66.4±12.1	65.2±12.6	<0.001

Adequacy				
Breakfast (0–10)	8.7±3.0	9.3±2.2	8.1±3.4	<0.001
Mixed grains (0–5)	2.6±2.2	2.7±2.2	2.4±2.2	<0.001
Total fruits (0–5)	2.5±2.2	2.5±2.3	2.5±2.2	0.386
Fresh fruits (0–5)	2.7±2.4	2.6±2.4	2.7±2.4	0.622
Total vegetables (0–5)	3.7±1.4	3.8±1.4	3.7±1.4	0.001
Fresh vegetables (0–5)	3.4±1.6	3.5±1.7	3.4±1.6	0.005
Meat, fish, egg, and legumes (0–10)	6.6±3.3	6.5±3.3	6.7±3.3	<0.001
Milk and its products (0–10)	2.6±4.1	2.5±4.0	2.8±4.2	<0.001

Moderation				
Saturated fatty acids (0–10)	8.8±2.9	9.0±2.6	8.6±3.1	<0.001
Sodium (0–10)	6.9±3.3	7.1±3.3	6.7±3.4	<0.001
Sweets and beverages (0–10)	9.5±1.8	9.5±1.8	9.4±1.9	0.140

Balance				
Carbohydrates (0–5)	1.9±2.1	1.8±2.0	2.1±2.1	<0.001
Total fat (0–5)	2.8±2.2	2.6±2.2	3.0±2.2	<0.001
Energy intake (0–5)	3.1±2.2	3.1±2.2	3.1±2.2	0.351

Values are presented as mean±standard deviation.

TE, total energy intake; KNHANES, Korea National Health and Nutrition Examination Survey.

1 Student t-test.

2 Information on healthy eating index and its components was available only for participants in the KNHANES 2013–2018.

**Table 3:** Associations of late eating with blood pressure control in adults with hypertension

Variables	Total (n)	BP control (%)	OR for BP control (95% CI)^1^		

			Model 1	Model 2	Model 3
Meal timing					
Early eaters	6,680	3,561 (53.3)	1.00 (reference)	1.00 (reference)	1.00 (reference)
Late eaters	6,681	3,228 (48.3)	0.82 (0.77, 0.88)	1.03 (0.94, 1.12)	1.02 (0.90, 1.15)

Gender					
Men	6,244	3,052 (48.9)	-	1.00 (reference)	1.00 (reference)
Women	7,117	3,737 (52.5)	-	0.86 (0.78, 0.95)	0.83 (0.72, 0.95)

Age (yr)	13,361	-		1.00 (0.99, 1.00)	1.00 (0.99, 1.01)

Shift working					
No	11,993	6,124 (51.1)		1.00 (reference)	1.00 (reference)
Yes	1,099	524 (47.7)		1.13 (0.96, 1.33)	1.14 (0.91, 1.44)

Current smoking					
No	11,010	5,721 (52.0)	-	1.00 (reference)	1.00 (reference)
Yes	2,211	991 (44.8)	-	1.12 (0.98, 1.28)	1.14 (0.94, 1.38)

Current drinking					
No	6,802	3,665 (53.9)	-	1.00 (reference)	1.00 (reference)
Yes	6,422	3,044 (47.4)	-	1.05 (0.95, 1.16)	1.06 (0.93, 1.21)

Walking, no. of days	13,087	-	-	1.02 (1.00, 1.03)	1.02 (1.00, 1.04)

Body mass index (kg/m^2^)	13,333	-	-	0.99 (0.98, 1.00)	0.99 (0.97, 1.01)

Comorbid status					
No	7,581	3,134 (41.3)	-	1.00 (reference)	1.00 (reference)
Yes	5,780	3,655 (63.2)	-	1.05 (0.96, 1.15)	1.05 (0.93, 1.18)

Adherence to antihypertensive medication					
Low	4,318	331 (7.7)	-	1.00 (reference)	1.00 (reference)
High	9,043	6,458 (71.4)	-	30.94 (27.05, 35.39)	187.86 (133.32, 264.70)

Total energy intake (100 kcal)	13,361	-	-	-	1.00 (0.99, 1.00)

Healthy Eating Index (10 score)	8,493	-	-	-	1.04 (0.99, 1.09)

Data include number of participants, proportion of controlled blood pressure within each group, and OR (95% CI) of late eating for BP control.

BP, blood pressure; OR, odds ratio; CI, confidence interval.

1 Model 1 shows crude ORs; Model 2 was adjusted for gender, age, shift work, smoking, drinking, walking, body mass index, comorbid status, and adherence to antihypertensive medication; Model 3 was additionally adjusted for total energy intake and healthy eating index plus the variables included in model 2.

**Table 4:** Associations of late eating with cardiometabolic risk factors in adults with hypertension

Variables	Cardiometabolic risk factors, mean±SD		Parameter estimate for cardiometabolic risk factors (p-value)	
	
	Early eaters	Late eaters	Crude model	Adjusted model^1^
Systolic blood pressure (mmHg)	134.2±16.8	133.6±16.6	−0.61 (0.035)	−0.38 (0.161)

Diastolic blood pressure (mmHg)	79.0±11.8	82.2±12.3	3.20 (<0.001)	0.19 (0.261)

BMI (kg/m^2^)	24.9±3.3	25.3±3.5	0.43 (<0.001)	0.13 (0.029)

Waist circumference (cm)	86.1±9.1	86.5±9.5	0.43 (0.008)	−0.09 (0.273)

Fasting glucose (mg/dL)	107.6±27.1	107.7±27.1	0.13 (0.790)	0.20 (0.643)

Hemoglobin A1c (%)	6.1±0.9	6.1±1.0	−0.02 (0.218)	0.01 (0.547)

Triglycerides (mg/dL)	150.4±100.0	165.1±134.8	14.70 (<0.001)	6.47 (0.003)

Total cholesterol (mg/dL)	188.4±38.7	191.9±39.1	3.50 (<0.001)	0.77 (0.253)

HDL cholesterol (mg/dL)	48.2±12.0	48.1±11.9	−0.12 (0.569)	−0.23 (0.270)

SD, standard deviation; BMI, body mass index; HDL, high-density lipoprotein.

1 Adjusted models included gender, age, shift work, smoking, drinking, walking, BMI, comorbid status, and adherence to antihypertensive medication. For BMI, BMI was excluded in the adjusted model; for fasting glucose or hemoglobin A1c, antidiabetic treatment (drug use or insulin injection) was additionally added in the adjusted model; for blood lipid levels, lipid-lowering medication was additionally added in the adjusted model.
