# Supplementary material for: Late eating, blood pressure control, and cardiometabolic risk factors among adults with hypertension: results from the Korea National Health and Nutrition Examination Survey 2010–2018
Source: Epidemiol Health. 2021 Nov 24;43:e2021101. doi: 10.4178/epih.e2021101 (PMC8920743; doi:10.4178/epih.e2021101)
Supplement: Supplementary file 1 [file epih-43-e2021101-suppl.docx]

**Supplementary Material 1.** Association of late eating with cardiometabolic risk factors in hypertensive adults who reported that their intake on the recall day was similar to the amount of food they usually ate.

| **Variables** | **Cardiometabolic risk**  **factors (mean±SD)** | |  | **Parameter estimate for**  **cardiometabolic risk factors**  **(p-value)** | |
| --- | --- | --- | --- | --- | --- |
|  | **Early eaters** | **Late eaters** |  | **Crude model** | **Adjusted model** |
| Systolic blood pressure, mmHg | 134.3±16.9 | 133.8±16.8 |  | -0.55 (0.101) | -0.38 (0.229) |
| Diastolic blood pressure, mmHg | 78.4±11.8 | 81.5±12.2 |  | 3.10 (<0.001) | 0.24 (0.204) |
| Body mass index, kg/m^2^ | 24.8±3.2 | 25.2±3.5 |  | 0.39 (<0.001) | 0.12 (0.073) |
| Waist circumference, cm | 86.0±9.1 | 86.3±9.5 |  | 0.36 (0.049) | -0.06 (0.520) |
| Fasting glucose, mg/dL | 107.8±26.8 | 107.8±27.4 |  | 0.10 (0.918) | 0.16 (0.746) |
| Hemoglobin A1c, % | 6.1±1.0 | 6.1±1.0 |  | -0.02 (0.434) | 0.01 (0.422) |
| Triglycerides, mg/dL | 149.7±99.8 | 161.4±128.2 |  | 11.72 (<0.001) | 12.06 (0.013) |
| Total cholesterol, mg/dL | 187.9±38.9 | 191.2±38.7 |  | 3.33 (<0.001) | 1.08 (0.430) |
| HDL cholesterol, mg/dL | 48.2±11.9 | 48.1±11.7 |  | -0.03 (0.900) | -0.30 (0.501) |

Data are presented as mean ± SD and parameter estimate (p-value). ^1^Adjusted models included sex, age, shift work, smoking, drinking, walking, body mass index, comorbid status, and antihypertensive medication. For body mass index, body mass index was excluded in the adjusted model; for fasting glucose or hemoglobin A1c, antidiabetic treatment (drug use or insulin injection) was additionally added in the adjusted model; for blood lipid levels, lipid-lowering medication was additionally added in the adjusted model
